# Reduced Social Connectedness and Compassion Toward Close Others in Patients With Chronic Depression Compared to a Non-clinical Sample

**DOI:** 10.3389/fpsyt.2021.608607

**Published:** 2021-03-18

**Authors:** Artjom Frick, Isabel Thinnes, Stefan G. Hofmann, Sabine Windmann, Ulrich Stangier

**Affiliations:** ^1^Department of Psychology, Goethe University Frankfurt, Frankfurt, Germany; ^2^Department of Psychological and Brain Sciences, Boston University, Boston, MA, United States

**Keywords:** social connectedness, interpersonal closeness, prosocial motivation, compassion, compassionate love, chronic depression, persistent depressive disorder

## Abstract

Reduced social functioning in depression has been explained by different factors. Reduced social connectedness and prosocial motivation may contribute to interpersonal difficulties, particularly in chronic depression. In the present study, we tested whether social connectedness and prosocial motivation are reduced in chronic depression. Forty-seven patients with persistent depression and 49 healthy controls matched for age and gender completed the Inclusion of the Other in the Self Scale (IOS), the Compassionate Love Scale (CLS), the Beck Depression Inventory-II, and the Childhood Trauma Questionnaire. A Multivariate analysis of variance (MANOVA) with IOS and CLS as dependent variables revealed a highly significant difference between both groups. The IOS and the CLS-subscale Close Others were lower in persistent depression, whereas there was no difference in the CLS-subscale Strangers/Humanity. IOS and CLS-Close Others showed significant negative correlations with depressive symptoms. Connectedness to family members as measured by the IOS was negatively correlated with childhood trauma in patients with chronic depression. The results indicate that compassion and perceived social connection are reduced in depressed patients toward close others, but not to others in general. Implications for the treatment of depression are discussed.

## Introduction

Depression is associated with a low level of social integration and connectedness (1) and reduced social functioning (2). Possible explanations for the social retreat of depressed patients refer to decreased pleasure from social interactions due to reduced response from the social reward system [social anhedonia, (3)] and hypersensitivity to social rejection (4), or dysfunctional interpersonal behaviors, such as excessive reassurance seeking or negative feedback seeking (5).

According to the social identity theory, the impairment of interpersonal relationships and social isolation affects the attachment to close others as well as the belonging to groups, resulting in a loss of social connectedness (6, 7). A recent longitudinal study which used objective indicators of social connectedness demonstrated that there are strong bidirectional associations between social disconnectedness and symptoms of depression (8). However, the perception of belonging to others, rather than objective social interaction, may be the component of social connectedness most relevant to the development and maintenance of depression (1, 7). For example, social connectedness is associated with increased motivation to make contact with other people (9) and may be a mediator of the positive effects of social competence and social support on mental health (10).

In addition, impairment of prosocial motivation may also affect social functioning in depressed individuals. This may be closely related to the reduction of perceived social connectedness. According to Batson et al. (11), prosocial motivation can be based on altruistic motives such as empathic concern or compassion. For instance, there is evidence that empathy is reduced during major depressive episodes (12). However, mixed results have been found with respect to prosocial motivation when using the Prisoner's Dilemma to investigate the link between depression and prosocial motivation (13).

A prominent feature of depression, although not specific to depression alone, is a self-critical attitude (14). Opposed to self-criticism is self-compassion, which entails the attitude to be “open to and moved by one's own suffering, experiencing feelings of caring and kindness toward oneself and taking an understanding non-judgmental attitude toward one's inadequacies and failures, and recognizing that one's experience is part of the common human experience” (15). A lack of self-compassion is a strong predictor of depressive symptoms in the general population (16) and in depressed patients (17), and self-compassion is significantly reduced in individuals with current (18, 19) as well as remitted depression (19). However, these studies have focused on major depressive disorder.

Although previous research demonstrated that *self*-compassion is reduced in depression, research on the role of compassion *toward others* in depression is sparse. A recent longitudinal study on the relationship between dispositional compassion and depressive symptoms found that among adolescents and young adults, high levels of dispositional compassion predicted lower depression, whereas conversely, depression was not likely to influence compassion (20). However, depressive symptoms were only mild and non-clinical in the sample studied. Another recent longitudinal study indicated that the experience of depressive episodes may even trigger increased compassion at a later time (21), possibly by inducing posttraumatic growth and compassionate identification with the suffering of others. However, during an acute depressive episode, prosocial constructs such as empathy appear to be impaired compared with healthy control subjects (22). So far, it is unclear whether dispositional compassion is altered in chronically depressed patients. According to the above-mentioned studies, on the one hand it could be assumed that patients with chronic depression develop a stronger dispositional compassion in the sense of posttraumatic growth due to their often long history of depression. On the other hand, it could be speculated that the acute symptoms during chronic depression tend to reduce compassion.

As compared to the episodic form of depression, Persistent Depressive Disorder (PDD) is associated with stronger impairment in life and higher social and economic costs (23). Although it has often been hypothesized that a chronic course of depression may be explained by dysfunctional interpersonal patterns, there is a lack of research supporting this assumption. Previous studies indicate, however, that chronic depression is characterized by a dysfunctional interpersonal style, as compared to patients with episodic depression (24, 25). Impaired social cognition, either in terms of a mood-congruent interpretive bias (26) or in terms of preoperational thinking (27), has been highlighted as a risk factor for the development of depressive symptomatology. In this context, indirect evidence for a relationship between impaired social cognition and a hostile and overly submissive interpersonal style has been discussed (26). Furthermore, preoperational thinking appears to mediate the association between adverse childhood experiences and a hostile interpersonal style in depressed patients (28). In particular, it has been shown that early emotional neglect, abuse, and rejection during childhood are important risk factors to interpersonal difficulties in chronic depression (29). However, up to now there is little research on the role of social connectedness and compassion in chronic depression. The present study aims at reducing this gap. We hypothesized that compared to healthy controls, patients with chronic depression report significantly lower social connectedness and less compassion. Since there is some evidence for gender differences in prosociality (13, 30, 31) and social connectedness (32), we hypothesized that women report higher compassion than men, and differ from men with respect to social connectedness. In addition, we explored whether social connectedness and compassion are significantly correlated, and whether both are related to severity of depressive symptoms and self-reported childhood adversity.

## Materials and Methods

### Participants and Recruitment

As part of the *MeCBT* study (33) we recruited 47 patients with PDD according to the DSM 5. These patients were compared to 49 control subjects without mental disorders who were recruited outside the project. A total of 35 women and 12 men aged 25–69 (*M* = 50.34; *SD* = 11.39) were in the group of chronically depressive patients, and 34 women and 15 men aged 27–69 (*M* = 50.06; *SD* = 12.81) were in the healthy control sample (see Supplementary Table 1 for more details). Healthy control subjects were recruited via public social media as well as via notices in public places. Interested participants registered by e-mail. If participants gave their written informed consent, a screening interview was conducted by telephone using the *Patient Health Questionnaire* [German version by Löw et al. (34)] to check for a current mental disorder. The interview was conducted by psychology master's degree candidates who had received specific training. If participants met inclusion criteria, they were provided a link to an online survey in which they filled out demographic information and completed the *Beck Depression Inventory-II* [BDI-II; German version by Hautzinger et al. (35)] and the questionnaires on compassion and social connectedness. The same questionnaires had been filled out on the same platform by the chronically depressed participants of the MeCBT study at baseline assessment. The survey included a mechanism to check for completion of the survey. The IOS-Item *romantic partner* was an exception and participants could leave this item blank if they currently had no romantic partner. The participants of the healthy control group received a compensation of €40 for participation. The two samples were matched for age and gender.

### Outcome Measures

#### Social Connectedness

As a proxy to social connectedness in the sense of *interpersonal closeness*, we used the *Inclusion of Other in the Self Scale* (IOS) developed by Aron et al. (36). The IOS is a pictorial measure that provides seven images showing two circles overlapping to different degrees, and the participant is asked to select the one image that best represents the relationship between him- or herself and a specified other person. It shows good psychometric properties, including convergent, discriminant and predictive validity (36). Aron et al. reported an overall retest-reliability of *r* = 0.83, and retest-reliabilities between *r* = 0.85 and *r* = 0.87 for single items (36). The IOS is efficient and valid in measuring relationship quality (37) and has been shown to predict helping behavior better than empathy (38). In the present study, the IOS was used to assess the extent of connectedness to five different groups of people: With (1) a partner, (2) family, (3) friends, (4) acquaintances, and (5) people in general.

#### Compassion

We used the *Compassionate Love Scale* (CLS) by Sprecher and Fehr (39) to assess compassion for others. According to Sprecher and Fehr, the construct *Compassionate Love* refers to *Agape*, one of the six love styles described by J. A. Lee (40). Agape is rooted in the occidental philosophy and is defined as altruistic love directed toward others. The CLS contains 21 items to be rated on a seven-point Likert scale ranged from 1 (not at all true of me) to 7 (very true of me). It exists in two versions: (a) compassion toward close others (friends, family) and (b) compassion toward strangers or all humanity. In the present study both versions were used. The CLS showed high internal consistency of α = 0.95 for both versions (39). However, no retest-reliability has been reported for the CLS and convergent validity has not yet been researched extensively. Moreover, the content validity, at least of a part of the scale, has been questioned recently (41, 42).

#### Childhood Adversity

The *Childhood Trauma Questionnaire* [CTQ; German version by Klinitzke et al. (43)] was used to assess childhood adversity in depressed patients. The CTQ is a self-report measure with good internal consistency and construct validity (43). Due to organizational restrictions, we did not administer the CTQ in the healthy control group. We added data from other studies for a representative sample as well as for a healthy sample in the Methods section to interpret the results for the chronically depressed patients (see also Supplementary Table 2 for more details).

#### Depressive Symptoms

The German version of the BDI-II (35) was used for the assessment of self-reported severity of depressive symptoms. The BDI-II has been shown to be a largely objective, reliable (internal consistency α ≥ 0.84), and valid instrument for assessing depressive symptoms (44).

### Statistical Analysis

For the main statistical analysis, we employed a two-factorial MANOVA. Factor 1 was *Group* and consisted of two levels (mentally healthy vs. PDD affected participants), factor two was *Gender*. The dependent variables were the scores in the Compassionate Love Scale - Close others, the Compassionate Love Scale - Strangers/All of Humanity, and the IOS-Items *family, friends, acquaintances* and *people in general*. IOS connectedness to a *romantic partner* was analyzed in a separate analysis of variance for all participants who filled out the item (i.e., were in a partnership, *N* = 75). The IOS item “romantic partner” was completed less frequently by the chronically depressed patients (*N* = 30) than by healthy controls (*N* = 45). We think that the reduced number of romantic partners may be representative for patients with chronic depression. However, due to this difference it is difficult to compare both groups with respect to the “romantic partner” item and results regarding this item should be considered with more caution than the other analyses. Bivariate correlations (*Pearson's r)* between age, BDI-II, CLS, and IOS scales were exploratively analyzed separately for the two groups. Additionally, among the group of chronically depressed patients, we examined the correlations between the above variables and the CTQ.

## Results

The MANOVA test using Pillai's Trace showed significant main effects of Group (*F*_(6, 87)_ = 12.05, *p* < 0.001, $\eta_{\text{p}}^{2}$ = 0.29) and Gender (*F*_(6, 87)_ = 3.43, *p* = 0.004, $\eta_{\text{p}}^{2}$ = 0.19) and a significant interaction effect of Group^*^Gender (*F*_(6, 87)_ = 2.41, *p* = 0.034, $\eta_{\text{p}}^{2}$ = 0.14) on CLS and IOS scales.

**Figure 1:**
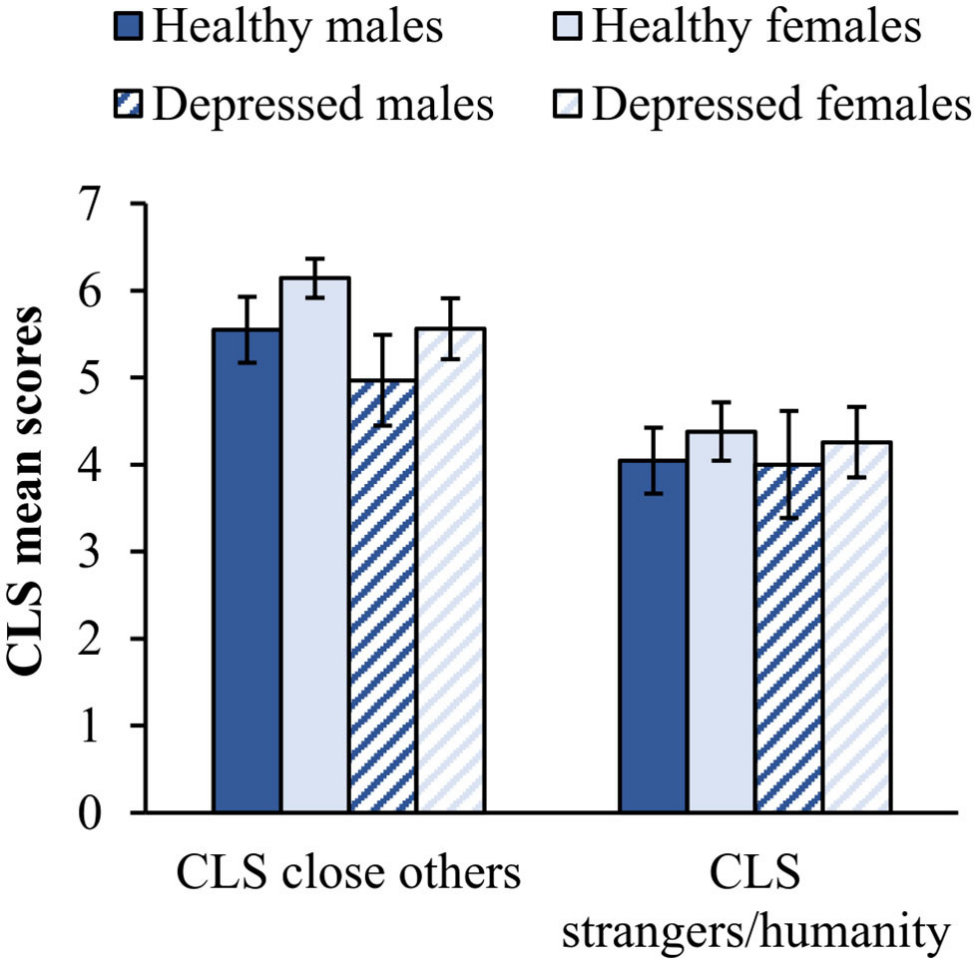
Mean CLS scores by Group and Gender. Error bars represent 95% confidence intervals. Healthy males *N* = 15, Healthy females *N* = 34, Depressed males *N* = 12, and Depressed females *N* = 35.

Univariate analyses (Table 1) showed no significant main Group effect (healthy controls: *M* = 4.28, *SD* = 1.05; patients with PDD: *M* = 4.19, *SD* = 1.18) or interaction effect of Gender by Group on compassion toward strangers/all humanity, as reflected in the CLS scores (see Figure 1 for CLS means by Group and Gender). However, compared to the chronically depressed patients (*M* = 5.41, *SD* = 1.05), healthy individuals (*M* = 5.96, *SD* = 0.74) had significantly higher compassion toward close others on the CLS scale, and significantly higher social connectedness on the IOS scale with their romantic partners (*M* = 4.98, *SD* = 1.63 vs. *M* = 3.43, *SD* = 1.83), family members (*M* = 5.06, *SD* = 1.23 vs. *M* = 3.15, *SD* = 1.84), friends (*M* = 4.00, *SD* = 1.14 vs. *M* = 3.36, *SD* = 1.57), acquaintances (*M* = 2.92, *SD* = 1.06 vs. *M* = 2.32, *SD* = 1.09), and people in general (*M* = 2.59, *SD* = 1.08 vs. *M* = 2.23, *SD* = 1.36; see Table 1 for *F*-values and effect sizes).

**Table 1:** Results of main and interaction effects of Group and Gender using univariate Analyses of Variance.

	**Group**		**Gender**		Group^*^Gender	
	***F***	***$\eta_{\text{p}}^{2}$***	***F***	***$\eta_{\text{p}}^{2}$***	***F***	***$\eta_{\text{p}}^{2}$***
CLS strangers/humanity^a^	0.11	< 0.01	1.34	0.01	0.02	< 0.01
CLS close others^a^	8.48^**^	0.08	8.82^**^	0.09	0.00	0.01
IOS romantic partner^b^	18.85^**^	0.21	1.85	0.03	3.67	0.05
IOS family^a^	32.09^**^	0.26	1.70	0.02	0.27	< 0.01
IOS friends^a^	14.34^**^	0.14	6.94^*^	0.07	10.88^**^	0.11
IOS acquaintances^a^	16.98^**^	0.16	0.71	0.01	11.66^**^	0.11
IOS people in general^a^	6.45^*^	0.07	2.19	0.02	6.72^*^	0.07

a *F(1, 92)*,

b *F(1, 71)*;

* *p < 0.05*;

** *p < 0.01; $\eta_{\text{p}}^{2}$ = partial eta-squared*.

**Figure 2:**
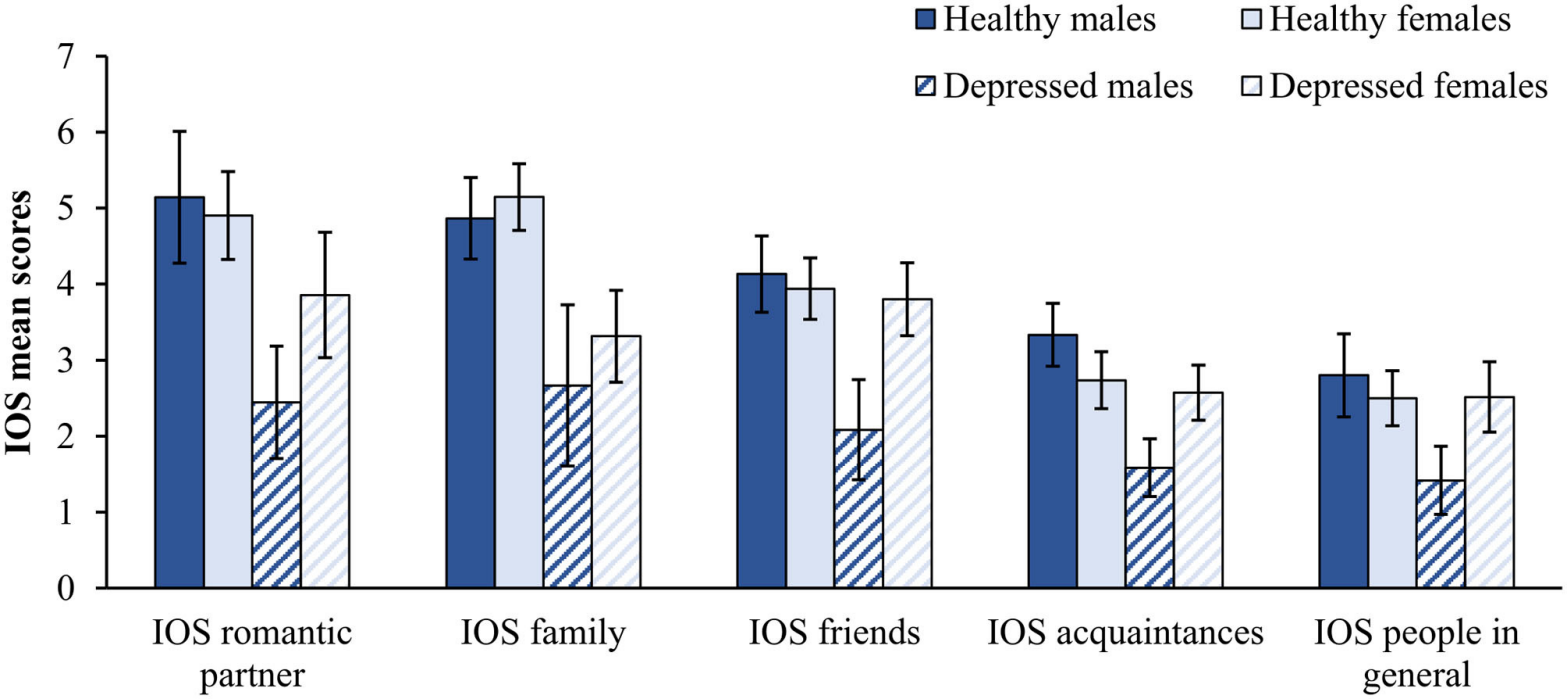
Mean IOS scores by Group and Gender. Error bars represent 95% confidence intervals. IOS romantic partner: Healthy males *N* = 14, Healthy females *N* = 31, Depressed males *N* = 9, Depressed females *N* = 21. Other IOS scales: Healthy males *N* = 15, Healthy females *N* = 34, Depressed males *N* = 12, and Depressed females *N* = 35.

In the total sample, women (*M* = 5.85, *SD* = 1.05) reported significantly more compassion toward close others than men on the CLS scale (*M* = 5.26, *SD* = 1.70), and higher connectedness with friends on the IOS scale (*M* = 3.87, *SD* = 1.33 vs. *M* = 3.22, *SD* = 1.48; see Table 1 for *F*-values and effect sizes). With regards to the latter, there was also a significant interaction effect of Group by Gender on social connectedness toward friends, acquaintances, and people in general (see Table 1 for *F*-values, Figure 2 for means and Supplementary Table 1 for details on descriptive statistics). Healthy men reported slightly higher values than healthy women for friends, acquaintances, and people in general, but depressed men reported significantly lower values than depressed women. Furthermore, while depressed women reported approximately the same level of social connectedness toward friends, acquaintances, and people in general as their healthy counterparts, depressed men reported significantly less social connectedness to all groups of people than healthy men. However, both, depressed women and men reported reduced social connectedness toward romantic partners and family members compared to their healthy counterparts (Figure 2).

Patients with PDD reported higher levels of childhood adversity (CTQ total score: *M* = 53.17, *SD* = 16.36) compared to a representative sample (43) as well as a healthy control group from a recent other study (45), especially regarding emotional abuse [*M* = 13.72, *SD* = 5.77 vs. *M* = 6.49, *SD* = 2.60 (43) and *M* = 7.0, *SD* = 3.5 (45), respectively] and emotional neglect *M* = 16.40, *SD* = 5.34 vs. *M* = 10.05, *SD* = 4.23 (43) and *M* = 8.3, *SD* = 3.1 (45), respectively; see Supplementary Table 2 for details]. Overall childhood adversity was significantly negatively correlated with connectedness to family members (Table 2). On the CTQ subscale level, IOS family correlated most strongly with emotional neglect, *r*(45) = −0.541, *p* < 0.001, emotional abuse, *r*(45) = −0.409, *p* < 0.01, and physical abuse, *r*(45) = −0.380, *p* < 0.01 (for details see Supplementary Table 3). Among the chronically depressed, compassion toward close others was significantly positively correlated with connectedness toward all groups of people except romantic partners. Compassion toward people in general was slightly positively correlated with connectedness toward more distant groups of people (friends, acquaintances, people in general). In the healthy control group, there was only a significant positive correlation between compassion toward close others and connectedness with family members, as wells as a significant negative correlation between compassion toward people in general and connectedness with a partner (Table 2). In the group of chronically depressed patients (BDI-II: *M* = 29.94, *SD* = 9.17; in total sample: *M* = 16.36, *SD* = 14.97), there was no correlation between severity of depressive symptomatology and connectedness or compassion. However, within the healthy control group (BDI-II: *M* = 3.35, *SD* = 3.14), there were a significant negative correlation between depression and connectedness with a romantic partner, and low or non-significant negative correlations between depression and connectedness with the other groups of people (Table 2).

**Table 2:** Bivariate correlations (Pearson's r) between childhood adversity (depression group only), age severity of depression, compassion and social connectedness in the healthy control group (above the diagonal) and in the group of chronically depressed patients (below the diagonal).

	**CTQ**	**Age (years)**	**BDI-II**	**CLS CO**	**CLS S/H**	**IOS RP**	**IOS FAM**	**IOS FR**	**IOS ACQ**	**IOS PG**
Age (years)	0.118	.	0.043	0.261	0.362^*^	−0.297^*^	−0.107	−0.209	−0.241	−0.040
BDI-II	0.239	−0.078	.	0.156	0.089	−0.385^**^	−0.103	−0.187	−0.161	−0.148
CLS CO	−0.053	−0.098	−0.133	.	0.547^**^	−0.083	0.299^*^	0.119	0.108	0.104
CLS S/H	0.168	−0.172	0.128	0.657^**^	.	−0.341^*^	−0.137	−0.112	−0.096	0.157
IOS RP	−0.017	0.167	−0.129	0.249	0.062	.	0.477^**^	0.584^**^	0.587^**^	0.245
IOS FAM	−0.470^**^	−0.216	−0.090	0.306^*^	0.071	0.337	.	0.551^**^	0.468^**^	0.349^*^
IOS FR	−0.058	−0.131	0.082	0.350^*^	0.223	0.400^*^	0.395^**^	.	0.867^**^	0.612^**^
IOS ACQ	−0.035	0.147	0.022	0.428^**^	0.330^*^	0.407^*^	0.356^*^	0.749^**^	.	0.737^**^
IOS PG	−0.003	0.138	−0.114	0.312^*^	0.220	0.396^*^	0.265	0.492^**^	0.658^**^	.

* *p < 0.05*;

** *p < 0.01; CTQ, Childhood Trauma Questionnaire; BDI-II, Beck Depression Inventory II; CLS CO, close others; CLS S/H, CLS strangers/humanity; IOS RP, IOS romantic partner; IOS FAM, IOS family; IOS FR, IOS friends; IOS ACQ, IOS acquaintances; IOS PG, IOS people in general*.

*N = 30 for IOS romantic partner and N = 47 for all other variables in the chronic depression group; N = 45 for IOS romantic partner and N = 49 for all other variables in the healthy control group*.

The lack of correlations between depression severity and social connectedness encouraged us to explore possible interactions with gender (see Supplementary Table 1 for details on descriptive statistics). In depressed men, severity of depression was correlated only with connectedness with a romantic partner (*r*(7) = 0.681, *p* < 0.05), whereas there were no significant correlations in depressed women. No significant correlations were also found in healthy men, while there was a significant moderate correlation between severity of depression and connectedness with a romantic partner in healthy women (*r*(29) = 0.413, *p* < 0.05). However, due to the small number of participants within the subsamples, these results should be interpreted with great caution.

## Discussion

Our hypothesis that patients with chronic depression would report lower perception of social connectedness as compared to healthy controls was supported by significant differences in the IOS scale. This is consistent with longitudinal data indicating bi-directional correlations between depressive symptoms and objective indicators of social disconnectedness, such as the frequency of social interactions, as well as the perception of social isolation during episodes of major depression (8). Although our cross-sectional design does not allow for causal conclusions, the results confirm the importance of perceived social disconnectedness and severe social impairment in social functioning among chronically depressed patients (2). Within the context of social identity theory, social relationships structure the individuals' self-concept and behavior (7). Thus, the perception of distorted relationships to others, low self-esteem, and reduced motivation to sustain social relationships may build a negative spiral (46), which may also contribute to the maintenance of depressed mood in persistent depressive disorder.

Contrary to our expectations and inconsistent with results from the longitudinal study by Santini et al. (8), social connectedness was not related to the severity of depressive symptoms within depressed patients. A possible reason for this may be the relatively high homogeneity of depression scores within the chronic depression group. Studies with larger samples are needed to provide more conclusive evidence. Furthermore, the link between social connectedness and depressive symptoms may be complicated by moderating variables such as prosocial behavior, in the sense that positively valued social interactions may be required to maintain interpersonal connectedness.

While we found significant differences between chronically depressed patients and healthy controls in all IOS subscales (i.e., romantic partner, family, friends, acquaintances, and people in general), a differential pattern occurred with respect to compassion. On the one hand, patients with chronic depression also reported significantly reduced compassion to close others. This result is consistent with findings of reduced dispositional compassion (20) and reduced empathy - as an integral part of compassion - in depression (12). On the other hand, and contrary to our expectations, compassion was not impaired toward strangers or other humans in general. A possible explanation of this discrepancy may be that compassion, as defined by “the sensitivity to suffering in self and others with a commitment to try to alleviate and prevent it” [(47), p. 2260], may affect the perception of social connectedness more in relationships to close others than to strangers.

Given the relationship between compassion and empathy, especially *empathic concern* (48), our findings can be related - with some caution - to existing research on the relationship between depression and empathy. *Distress tolerance*, the “ability to tolerate difficult emotions in oneself when confronted with someone else's suffering” is considered an aspect of compassion (41) and may reflect low trait *personal distress*, another facet of empathy. Considering previous findings of increased personal distress (49, 50) and our present findings of reduced compassion in patients with PDD, it could be hypothesized that patients with PDD experience other people's negative experiences as distressing rather than responding to them compassionately (i.e., with emotional concern). However, the assumption of reduced empathic concern would be in contrast to findings that suggest that empathic concern is either not reduced (49, 51) or is even increased (50) in patients with PDD. Moreover, our findings of reduced compassion in PDD cannot be validly reconciled with other studies' findings of increased personal distress (as a correlate of reduced compassion) because the CLS contains few items regarding distress tolerance (41) and correlates only weakly with personal distress (48). Yet, our results may be regarded consistent with Guhn et al. (52), who found markedly blunted emotional reactivity toward negative stimuli, such as others' suffering, in patients with PDD as compared with patients with recurrent depression or healthy controls.

It should be noted that Neugebauer et al. (21) observed in a longitudinal study that episodes of major depression were subsequently followed by increased altruism as assessed by a scale comprising compassion, social love, and human engagement. Interestingly, in our patient sample, reduced social connectedness was significantly correlated with reduced compassion toward close others, but not toward strangers or other humans in general. Importantly, reduced connectedness toward family members was also associated with self-reported childhood adversity, in particular with emotional abuse and emotional neglect. Thus, it is possible that the effect of childhood adversity as a risk factor for chronic depression may be meditated by the impairment of social connectedness in close relationships, rather than in relationships to others in general (53). By contrast, in chronically depressed patients, compassion toward close others or strangers was unrelated to childhood adversity. Thus, we could not confirm the findings of Lim and DeSteno (54), who found a significant positive correlation of life adversity with compassion toward others. However, it should be noted that we assessed severe and traumatic adversities in childhood, which may not have the same beneficial effects on prosocial attitudes and motivation in terms of posttraumatic growth. Further, our findings are consistent with a recent study that found an association between emotional abuse with loneliness, with the association mediated by increased rejection sensitivity (45).

There was a significant difference between chronically depressed patients and healthy controls with respect to gender effects on social connectedness. For both genders, depression was associated with a reduced degree of social connectedness, but the difference was significantly greater for men than for women. Thus, chronic depression was stronger associated with reduced social connectedness in men than in women. This may relate to the findings that men have less stable relationships with friends and acquaintances (55), weaker networks of social support (56) and less resources of emotional support in their social environment (57) than women. Following a divorce, men experience longer phases of psychological distress (58) and are at greater risk of suicide (59). Thus, although the risk of developing depression is higher in women (60), the effect of depression on social connectedness may be more serious in men.

There are several limitations to be noted. First, the use of cross-sectional data does not allow to make statements about the causality of the findings. Further studies with longitudinal study designs would be necessary to investigate possible causalities and, if applicable, their direction. Second, the reduced statistical power due to the small sample may increase the likelihood of false negative results. Thus, our findings warrant a replication in a larger sample. Third, the specificity of the results for chronic depression needs to be tested in future studies by including patients with major depressive disorder and other clinical conditions. Fourth, a multi-method approach using clinical ratings in addition to self-report measures may increase the validity of the findings. While the validity of the IOS has been repeatedly demonstrated (37), the compassionate love scale needs further testing. Hence, findings regarding compassion should be interpreted with caution.

To conclude, we found evidence for reduced social connectedness and compassion toward close others in chronically depressed patients, which should be addressed in the treatment. Based on findings that patients with chronic depression experience lower social integration, less social support (61), and smaller social networks (62), it can be speculated that social connectedness and belongingness are particularly impaired in patients with chronic depression, as compared to episodic depression. As for depressed patients in general, reduced social connectedness may also correspond with reduced empathic response to positive affect (51), impaired social cognition (26, 27), and social anhedonia (3). Psychological interventions for chronic depression should therefore target interpersonal problems. In line with this recommendation, Cognitive Behavioral Analysis System of Psychotherapy (CBASP) has proven to be effective in the treatment of chronic depression (63). In addition, meditation techniques focusing on loving kindness (64, 65) have also shown promising results. Thus, besides using cognitive and behavioral interventions focusing of specific interpersonal deficits, the enhancement of prosocial motivation and positive affect by combining CBT with metta meditation may also be an effective approach in the treatment of chronic depression (33).

## Data Availability Statement

The datasets presented in this article are not readily available because of patient confidentiality and participant privacy. Requests to access the datasets should be directed to frick@psych.uni-frankfurt.de.

## Ethics Statement

The studies involving human participants were reviewed and approved by Ethics Committee of the Faculty of Medicine at Goethe University Frankfurt (MeCBT study, chronically depressed patients); Ethics Committee of the Faculty of Psychology and Sports Sciences at Goethe University Frankfurt (healthy control group). The patients/participants provided their written informed consent to participate in this study.

## Author Contributions

US conceived the study and obtained ethical approval. AF, IT, SH, and SW contributed to conception and design of the study. IT and AF coordinated recruitment and data collection. AF performed the statistical analysis. AF and US drafted the article. SH and SW contributed to the review of literature and proofread the manuscript. AF edited the final manuscript. All authors contributed to manuscript revision, read, and approved the submitted version.

## Conflict of Interest

SH was supported by the James S. McDonnell Foundation 21st Century Science Initiative in Understanding Human Cognition - Special Initiative. The remaining authors declare that the research was conducted in the absence of any commercial or financial relationships that could be construed as a potential conflict of interest.

## Abbreviations

BDI-II: Beck Depression Inventory II
CLS: Compassionate Love Scale
CTQ: Childhood Trauma Questionnaire
DSM-5, Diagnostic and Statistical Manual of Mental Disorders: Fifth Edition
IOS: Inclusion of Other in the Self scale
MANOVA: Multivariate analysis of variance
PDD: Persistent depressive disorder.
