# Supplementary material for: Reduced Social Connectedness and Compassion Toward Close Others in Patients With Chronic Depression Compared to a Non-clinical Sample
Source: Front Psychiatry. 2021 Mar 18;12:608607. doi: 10.3389/fpsyt.2021.608607 (PMC8012512; doi:10.3389/fpsyt.2021.608607)
Supplement: Supplementary file 1 [file Table_1.docx]

| Supplementary Table 1. *Descriptive statistics: Sample sizes, means and standard deviations for all participants, separately by Group, Gender and Group*Gender* | | | | | | | | | |
| --- | --- | --- | --- | --- | --- | --- | --- | --- | --- |
|  | All participants | Patients with PDD | Healthy controls | Men | Women | Men with PDD | Healthy men | Women with PDD | Healthy women |
| *N* | 96 | 47 | 49 | 27 | 69 | 12 | 15 | 35 | 34 |
| *N* IOS romantic partner | 75 | 30 | 45 | 23 | 52 | 9 | 14 | 21 | 31 |
|  | *M (SD)* | | | | | | | | |
| Age (in years) | 50.20 (12.07) | 50.34 (11.39) | 50.06 (12.81) | 49.11 (13.01) | 50.62 (11.76) | 51.83 (12.55) | 46.93 (13.38) | 49.83 (11.12) | 51.44 (12.51) |
| BDI-II total score | 16.36 (14.97) | 29.94 (9.17) | 3.35 (3.14) | 16.56 (16.53) | 16.29 (14.45) | 33.5 (8.40) | 3.00 (2.98) | 28.71 (9.21) | 3.50 (3.24) |
| CLS close others | 5.69 (0.94) | 5.41 (1.05) | 5.96 (0.74) | 5.29 (0.87) | 5.85 (0.93) | 4.97 (0.93) | 5.55 (0.75) | 5.56 (1.06) | 6.14 (0.67) |
| CLS strangers/humanity | 4.24 (1.11) | 4.19 (1.18) | 4.28 (1.05) | 4.02 (1.12) | 4.32 (1.11) | 4.00 (1.09) | 4.04 (1.18) | 4.26 (1.22) | 4.38 (0.99) |
| IOS romantic partner | 4.36 (1.86) | 3.43 (1.83) | 4.98 (1.63) | 4.09 (1.98) | 4.48 (1.82) | 2.44 (1.13) | 5.14 (1.66) | 3.86 (1.93) | 4.90 (1.64) |
| IOS family | 4.13 (1.83) | 3.15 (1.84) | 5.06 (1.23) | 3.89 (1.83) | 4.22 (1.83) | 2.67 (1.87) | 4.87 (1.06) | 3.31 (1.83) | 5.15 (1.31) |
| IOS friends | 3.69 (1.39) | 3.36 (1.57) | 4.00 (1.14) | 3.22 (1.48) | 3.87 (1.33) | 2.08 (1.16) | 4.13 (0.99) | 3.80 (1.45) | 3.94 (1.20) |
| IOS acquaintances | 2.63 (1.11) | 2.32 (1.09) | 2.92 (1.06) | 2.56 (1.15) | 2.65 (1.10) | 1.58 (0.67) | 3.33 (0.82) | 2.57 (1.09) | 2.74 (1.11) |
| IOS people in general | 2.42 (1.23) | 2.23 (1.35) | 2.59 (1.08) | 2.19 (1.18) | 2.51 (1.24) | 1.42 (0.79) | 2.80 (1.08) | 2.51 (1.40) | 2.50 (1.08) |
| *PDD* = Persistent depressive disorder; *N* = number of individuals; *M* = mean; *SD* = standard deviation; *BDI-II* = Beck Depression Inventory II; *CLS* = Compassionate Love Scale; *IOS* = Inclusion of Other in the Self scale | | | | | | | | | |
