# Supplementary material for: Reduced Social Connectedness and Compassion Toward Close Others in Patients With Chronic Depression Compared to a Non-clinical Sample
Source: Front Psychiatry. 2021 Mar 18;12:608607. doi: 10.3389/fpsyt.2021.608607 (PMC8012512; doi:10.3389/fpsyt.2021.608607)
Supplement: Supplementary file 2 [file Table_2.DOCX]

| Supplementary Table 2. *Descriptive statistics: Means and standard deviations of the CTQ total score and CTQ subscales from the present study for all participants with PDD and separately by Gender; for a representative German sample; and for a healthy control group* | | | | | |
| --- | --- | --- | --- | --- | --- |
|  | Patients with PDD, present study | Depressed men, present study | Depressed women, present study | Representative sample, Klinitzke et al. (2011)^a^ | Healthy control group (HC_PDD_), Nenov-Matt (2020)^b^ |
| *N* | 47 | 12 | 35 | 2500 | 34 |
|  | *M (SD)* | | | | |
| Age (years) | 50.34 (11.39) | 51.83 (12.55) | 49.83 (11.12) | 50.6 (18.6) | 38.2 (12.3) |
| CTQ total score | 53.17 (16.36) | 51.33 (13.17) | 53.80 (17.45) | - | - |
| Emotional abuse | 13.72 (5.77) | 13.00 (6.16) | 13.97 (5.70) | 6.49 (2.60) | 7.0 (3.5) |
| Physical abuse | 7.55 (3.79) | 8.42 (4.12) | 7.26 (3.68) | 5.88 (2.18) | 5.7 (2.0) |
| Sexual abuse | 7.09 (3.72) | 5.25 (0.62) | 7.71 (4.12) | 5.45 (1.66) | 5.6 (1.6) |
| Emotional neglect | 16.40 (5.34) | 16.67 (4.83) | 16.31 (5.57) | 10.05 (4.23) | 8.3 (3.1) |
| Physical neglect | 8.40 (2.77) | 8.00 (2.49) | 8.54 (2.88) | 8.10 (3.00) | 6.5 (2.6) |
| ^a ­­­^Klinitzke G, Romppel M, Häuser W, Brähler E, Glaesmer H. Die deutsche Version des Childhood Trauma Questionnaire (CTQ) - psychometrische Eigenschaften in einer bevölkerungsrepräsentativen Stichprobe. *Psychother Psychosom Med Psychol* (2012) **62**:47–51. doi:10.1055/s-0031-1295495  ^b^ Nenov-Matt T, Barton BB, Dewald-Kaufmann J, Goerigk S, Rek S, Zentz K, et al. Loneliness, Social Isolation and Their Difference: A Cross-Diagnostic Study in Persistent Depressive Disorder and Borderline Personality Disorder. *Front. Psychiatry* (2020) **11**:608476. doi:10.3389/fpsyt.2020.608476  *PDD* = Persistent depressive disorder; *N* = number of individuals; *M* = mean; *SD* = standard deviation; *CTQ* = Childhood Trauma Questionnaire | | | | | |
