# Supplementary material for: Reduced Social Connectedness and Compassion Toward Close Others in Patients With Chronic Depression Compared to a Non-clinical Sample
Source: Front Psychiatry. 2021 Mar 18;12:608607. doi: 10.3389/fpsyt.2021.608607 (PMC8012512; doi:10.3389/fpsyt.2021.608607)
Supplement: Supplementary file 3 [file Table_3.docx]

| Supplementary Table 3. *Bivariate correlations (Pearson’s r) between age, severity of depressive symptoms, compassion, social connectedness and childhood adversity (depression group only) in the total sample (above the diagonal) and in the group of chronically depressed patients (below the diagonal).* | | | | | | | | | | | | | | |
| --- | --- | --- | --- | --- | --- | --- | --- | --- | --- | --- | --- | --- | --- | --- |
|  | Age (years) | BDI-II | CLS CO | CLS S/H | IOS RP | IOS FAM | IOS FR | IOS ACQ | IOS PG | CTQ | CTQ EA | CTQ PA | CTQ SA | CTQ EN |
| Age (years) | . | -.007 | .056 | .099 | -.124 | -.144 | -.161 | -.061 | .049 | . | . | . | . | . |
| BDI-II | -.078 | . | -.293^**^ | .015 | -.443^**^ | -.504^**^ | -.194 | -.253^*^ | -.182 | . | . | . | . | . |
| CLS CO | -.098 | -.133 | . | .594^**^ | .226 | .401^**^ | .319^**^ | .347^**^ | .265^**^ | . | . | . | . | . |
| CLS S/H | -.172 | .128 | .657^**^ | . | -.081 | .013 | .094 | .133 | .196 | . | . | . | . | . |
| IOS RP | .167 | -.129 | .249 | .062 | . | .521^**^ | .527^**^ | .562^**^ | .360^**^ | . | . | . | . | . |
| IOS FAM | -.216 | -.090 | .306^*^ | .071 | .337 | . | .491^**^ | .466^**^ | .324^**^ | . | . | . | . | . |
| IOS FR | -.131 | .082 | .350^*^ | .223 | .400^*^ | .395^**^ | . | .803^**^ | .550^**^ | . | . | . | . | . |
| IOS ACQ | .147 | .022 | .428^**^ | .330^*^ | .407^*^ | .356^*^ | .749^**^ | . | .696^**^ | . | . | . | . | . |
| IOS PG | .138 | -.114 | .312^*^ | .220 | .396^*^ | .265 | .492^**^ | .658^**^ | . | . | . | . | . | . |
| CTQ | .118 | .239 | -.053 | .168 | -.017 | -.470^**^ | -.058 | -.035 | -.003 | . | . | . | . | . |
| CTQ EA | -.014 | .197 | -.101 | .109 | .008 | -.409^**^ | -.085 | -.031 | .067 | .872^**^ | . | . | . | . |
| CTQ PA | .136 | .417^**^ | -.169 | .109 | -.290 | -.380^**^ | -.082 | -.002 | -.034 | .685^**^ | .493^**^ | . | . | . |
| CTQ SA | .233 | .125 | .084 | .151 | .102 | -.161 | .103 | .095 | .069 | .670^**^ | .480^**^ | .343^*^ | . | . |
| CTQ EN | .156 | .078 | -.026 | .072 | .049 | -.541^**^ | -.158 | -.169 | -.058 | .855^**^ | .719^**^ | .436^**^ | .423^**^ | . |
| CTQ PN | -.075 | .117 | .067 | .275 | .043 | -.144 | .116 | .057 | -.089 | .605^**^ | .365^*^ | .351^*^ | .332^*^ | .461^**^ |
| * *p* < 0,05; ** *p* < 0.01; *BDI-II* = Beck Depression Inventory II total score; *CLS CO* = close others; *CLS S/H* = CLS strangers/humanity; *IOS RP* = IOS romantic partner; *IOS FAM* = IOS family; *IOS FR* = IOS friends; *IOS ACQ* = IOS acquaintances; *IOS PG* = IOS people in general; *CTQ* = Childhood Trauma Questionnaire total score; CTQ EA = CTQ Emotional abuse; CTQ PA = CTQ Physical abuse; CTQ SA = CTQ Sexual abuse; CTQ EN = CTQ Emotional neglect; CTQ PN = CTQ Physical neglect | | | | | | | | | | | | | | |
| Note: *N* = 30 for IOS romantic partner and *N* = 47 for all other variables in the chronic depression group; *N* = 75 for IOS romantic partner and *N* = 96 for all other variables in the total sample | | | | | | | | | | | | | | |
